# Different Forms of TFF2, A Lectin of the Human Gastric Mucus Barrier: *In Vitro* Binding Studies

**DOI:** 10.3390/ijms20235871

**Published:** 2019-11-22

**Authors:** Franziska Heuer, René Stürmer, Jörn Heuer, Thomas Kalinski, Antje Lemke, Frank Meyer, Werner Hoffmann

**Affiliations:** 1 Institute of Molecular Biology and Medicinal Chemistry, Otto-von-Guericke University Magdeburg, Leipziger Str. 44, 39120 Magdeburg, Germany; 2 Institute of Pathology, Otto-von-Guericke University Magdeburg, 39120 Magdeburg, Germany; 3 Department of Surgery, Otto-von-Guericke University Magdeburg, 39120 Magdeburg, Germany

**Keywords:** glycoproteins, *H. pylori*, lectin, mucin, peptides

## Abstract

Trefoil factor family 2 (TFF2) and the mucin MUC6 are co-secreted from human gastric and duodenal glands. TFF2 binds MUC6 as a lectin and is a constituent of the gastric mucus. Herein, we investigated human gastric extracts by FPLC and identified mainly high- but also low-molecular-mass forms of TFF2. From the high-molecular-mass forms, TFF2 can be completely released by boiling in SDS or by harsh denaturing extraction. The low-molecular-mass form representing monomeric TFF2 can be washed out in part from gastric mucosa specimens with buffer. Overlay assays with radioactively labeled TFF2 revealed binding to the mucin MUC6 and not MUC5AC. This binding is modulated by Ca^2+^ and can be blocked by the lectin GSA-II and the monoclonal antibody HIK1083. TFF2 binding was also inhibited by Me-β-Gal, but not the α anomer. Thus, both the α1,4GlcNAc as well as the juxtaperipheral β-galactoside residues of the characteristic GlcNAcα1→4Galβ1→R moiety of human MUC6 are essential for TFF2 binding. Furthermore, there are major differences in the TFF2 binding characteristics when human is compared with the porcine system. Taken together, TFF2 appears to fulfill an important role in stabilizing the inner insoluble gastric mucus barrier layer, particularly by its binding to the mucin MUC6.

## 1. Introduction

TFF2 (formerly: “spasmolytic polypeptide”) belongs to the family of secretory trefoil factor family (TFF) peptides and mainly consists of two cysteine-rich TFF domains (total length: 106 amino acid residues) [1,2,3,4]. The major expression sites in humans are the gastric mucous neck and antral gland cells and duodenal Brunner’s glands, where it is co-secreted with the mucin MUC6 [5,6,7]. Of note, TFF2 mRNA transcript expression in the gastric fundus is not parallel with peptide localization and rather marks a progenitor of mucous neck cells [8,9]. Pathologically, TFF2 is expressed by the ulcer-associated cell lineage, e.g., in the ileum of patients with Crohn’s disease [10]. In the pig, TFF2 is also secreted by the exocrine pancreas [11].

One of the major differences between human and porcine TFF2 is that the majority of human TFF2 is N-glycosylated, whereas porcine TFF2 lacks an N-glycosylation site [3,11]. As a hallmark, human gastric TFF2 contains an N-linked monofucosylated N,N’-diacetyllactosediamine (LacdiNAc) oligosaccharide [12]. The unusual LacdiNAc moiety is also carried by the gastric mucin MUC5AC and is recognized by the *Helicobacter pylori* adhesin LabA [13]. This is one reason why *H. pylori* mainly adhere to the MUC5AC layer. TFF2 is a typical constituent of the gastric juice, and there is a dramatic diurnal variation in the TFF2 concentration with a maximum between 05:00 and 07:00 [14]. Of note, N-glycosylation also varied diurnally with a maximum between 17:00 and 23:00 [14].

In humans as well as in pigs, TFF2 is associated with the gastric mucus fraction after size exclusion chromatography (SEC), probably due to lectin binding to the mucin MUC6 [15,16,17]. By the use of immunohistochemistry, TFF2 has also been clearly localized within the laminated array of gastric mucus [7]. Furthermore, TFF2 has been shown to affect the viscoelastic properties of gastric mucus *in vitro* and *in vivo* indicating interaction with gastric mucins [18,19]. The carbohydrate epitope recognized by a recombinant human TFF2 fusion protein was narrowed down in a porcine gastric mucus preparation to the peripheral GlcNAcα1→4Galβ1→4GlcNAcβ moiety [20]. Of note, the peripheral α1,4GlcNAc residue is exclusively limited to MUC6 secreting cells (i.e., gastric mucous and antral glands cells and duodenal Brunner’s glands). This α1,4GlcNAc residue at the non-reducing terminals of O-linked glycans is conserved during evolution from frog to human, and it is specifically recognized by the monoclonal antibody HIK1083 [21] as well as the lectin GSA-II from *Griffonia simplicifolia* [22,23,24]. α1,4-N-acetylglucosaminyltransferase (α4GnT) is the key enzyme for the synthesis of this unusual epitope. Remarkably, α1,4GlcNAc-capped MUC6 suppresses *H. pylori* growth, and a loss of the peripheral α1,4GlcNAc moiety in *A4GnT*-deficient mice results in differentiated-type adenocarcinoma of the stomach [25,26].

TFF2 is capable of supporting cell migration processes *in vitro* by a weak chemotactic effect, and there is a synergism with an epidermal growth factor [27,28]. Intragastric administration of TFF2-secreting *Lactococcus lactis* diminishes induced colitis in mice [29]. *Tff2*-deficient mice (*Tff2^KO^*) do not show a striking phenotype, they have only moderate morphological changes in the stomach, and they have increased susceptibility and accelerated progression to *Helicobacter*-induced gastritis [30,31,32]. However, TFF2 is also expressed in lymphoid organs, and in *Tff2^KO^* mice, the inflammatory and proliferative responses of immune cells were dysregulated [31,33]. Thus, TFF2 seems to have protective effects on mucous epithelia and also regulates inflammatory responses [2,4,28]. Other than strong lectin binding to MUC6, TFF2 has been reported to interact weakly also with β-integrin, DMBT1, PAR2, CXCR4, and CXCR7 [34,35,36].

Here, we investigated the TFF2 biosynthesis systematically in the human gastric mucosa, identified high- and low-molecular-mass forms by the use of SEC, and performed first binding studies of human mucin preparations with radioactively labeled TFF2. This is a further step towards understanding the role of TFF2 in the gastric and duodenal mucus barriers; these protective hydrogels have important functions at least for the secretion of hydrochloric acid (“viscous fingering”), adhesion of the human gastric and duodenal microbiota, absorption of nutrients, and drug delivery [37,38].

## 2. Results

### 2.1. Characterization of Human Gastric Extracts by SEC and Western Blot Analysis

**Figure 1:**
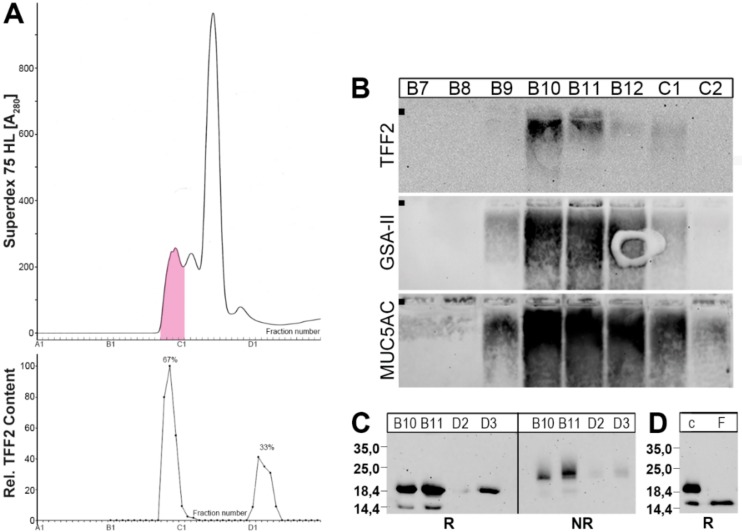
Analysis of human gastric extract (M_C_-577). (**A**) Elution profile after SEC on a Superdex 75 HL column as determined by absorbance at 280 nm (PAS-positive mucin fractions: pink). Underneath: Distribution of the relative trefoil factor family 2 (TFF2) content as determined by Western blot analysis under reducing conditions and semi-quantitative analysis of the typical 20 k-band intensities. (**B**) 1% agarose gel electrophoresis (AgGE) and subsequent Western blot analysis of the fractions B7-C2 concerning TFF2, MUC6 (lectin GSA-II), and MUC5AC, respectively. The start is marked with a dot on the left. (**C**) Fifteen percent SDS-PAGE and subsequent Western blot analysis of fractions B10/B11 and D2/D3 concerning TFF2 (R, NR: reducing and non-reducing conditions, respectively). The molecular mass standard is indicated left. (**D**) Digestion of fraction B12 with PNGase F and Western blot analysis concerning TFF2 (15% SDS-PAGE, reducing conditions); c, undigested control; F, sample digested with PNGase F.

When human gastric extracts were subject to SEC (Figure 1), TFF2 immunoreactivity appeared in two regions, i.e., in a periodic acid-Schiff (PAS)-positive high-molecular-mass peak and a low-molecular-mass peak (Figure 1A). The high-molecular-mass peak contained the mucins MUC5AC and MUC6 as detected after agarose gel electrophoresis (AgGE, Figure 1B), and under these conditions, TFF2 was clearly associated with mucins (Figure 1B). TFF2 immunoreactivity was strongly diminished after SDS-PAGE under non-reducing conditions when compared with reducing conditions (Figure 1C). TFF2 occurred mainly in an N-glycosylated form, and only to a minor extent in a non-glycosylated form, the latter could also be generated by digestion with peptide-N-glycosidase F (PNGase F) (Figure 1D).

**Figure 2:**
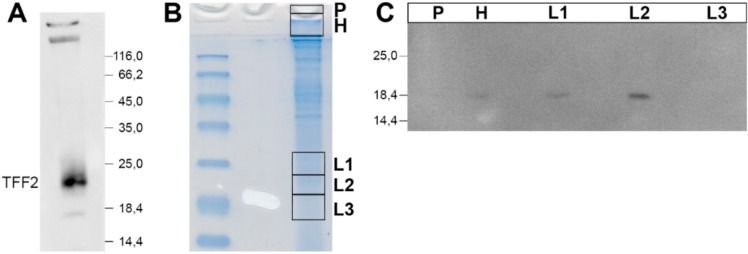
Relative TFF2 content in the high- and low-molecular-mass regions after non-reducing SDS-PAGE (Mc-577). (**A,B**) TFF2-containing fraction B12 after SEC of a human gastric extract (from Figure 1) was subjected to non-reducing 15% SDS-PAGE: Western blot analysis concerning TFF2 (A); Coomassie-stained part of the gel (B). Then, the high- (H) and low-molecular-mass regions (L1–L3) were cut out as indicated, proteins were eluted, and subjected to reducing SDS-PAGE. In addition, the remaining high-molecular-mass-samples not entering the gel were removed from the gel pockets (P). (**C**) Western blot analysis concerning TFF2 of P, H, and L1–L3 (reducing conditions).

Porcine gastric TFF2 has been shown to bind strongly to mucin, even after boiling in SDS (i.e., after non-reducing SDS-PAGE) [39]. To test if human gastric TFF2 still exists in high-molecular-mass forms after non-reducing SDS-PAGE (Figure 2A), samples were analyzed for their TFF2 content from the gel pocket, and the high- and low-molecular-mass regions, respectively (Figure 2B). TFF2 was mainly detectable in the low-molecular-mass region, only a little TFF2 was present in the high-molecular-mass region, and nothing in the pocket (Figure 2C).

**Figure 3:**
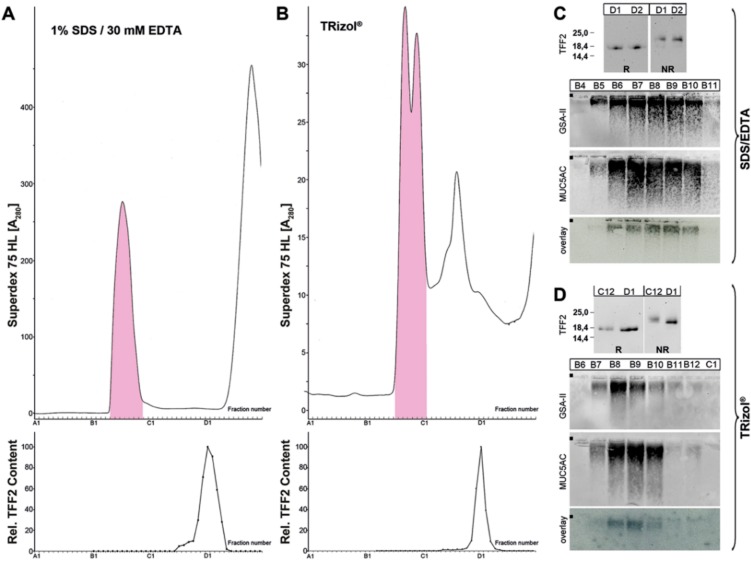
Analysis of human gastric extracts after boiling with SDS/EDTA (M_C_-406A) or after TRIzol^®^ extraction (M_C_-577), respectively. (**A**) High-molecular-mass TFF2 was purified via SEC from the human gastric corpus, similar to as shown in Figure 1. Then, the corresponding fractions (B6–B10) were boiled with 1% SDS + 30 mM EDTA and separated immediately on a Superdex 75 HL column again. Elution profile after SEC as determined by absorbance at 280 nm (PAS-positive mucin fractions: pink). Underneath: Distribution of the relative TFF2 content. (**B**) Alternatively, a human gastric corpus specimen was extracted with TRIzol^®^. Elution profile after SEC on a Superdex 75 HL column (PAS-positive fractions: pink). Underneath: Distribution of the relative TFF2 content. (**C**,**D**) Western blot analysis concerning TFF2 (15% SDS-PAGE), MUC6 (lectin GSA-II, 1% AgGE), and MUC5AC (1% AgGE), respectively, of the SDS/EDTA extract (**C**) or the TRIzol^®^ extract (**D**). Shown are also the hybridization signals (autoradiography) obtained after incubating the blots with ^125^I-labeled recombinant human non-glycosylated TFF2 (**C**) or porcine pancreatic TFF2 (**D**) (overlay assays). The start of the AgGE is marked with a dot on the left.

The binding of porcine gastric TFF2 to mucin is not only resistant to boiling in SDS, but also to extraction under harsh denaturing conditions (TRIzol^®^ reagent/pH 1) [39]. To compare the situation in both species, high-molecular-mass TFF2 was purified via SEC (similar as shown in Figure 1), then boiled in SDS/EDTA, and subject again to SEC (Figure 3A). Furthermore, human gastric specimens were extracted with TRIzol^®^ reagent and analyzed by SEC (Figure 3B). In both experiments, TFF2 could be released from the mucin fraction and appeared in a low-molecular-mass form only.

### 2.2. Stepwise Extraction of TFF2 from Human Gastric Mucosa

**Figure 4:**
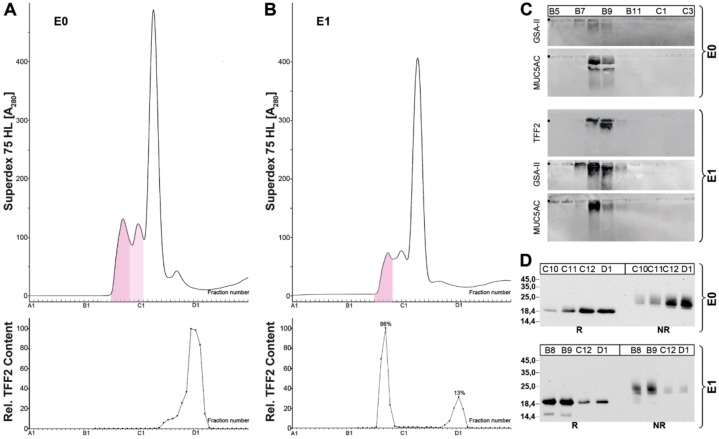
Stepwise extraction of a human gastric corpus specimen (M_C_-577) and analysis of the extracts E0 and E1. (**A,B**) Elution profiles of extract E0 (A) and E1 (B), respectively, after SEC on a Superdex 75 HL column as determined by absorbance at 280 nm (PAS-positive mucin fractions: pink). Underneath: Distribution of the relative TFF2 content. (**C**) 1% AgGE and subsequent Western blot analysis of the high-molecular-mass fractions B5-C3 concerning MUC6 (lectin GSA-II), MUC5AC, and TFF2. (**D**) 15% SDS-PAGE under reducing (R) or non-reducing conditions (NR) of low- and high-molecular-mass fractions and Western blot analysis concerning TFF2 (post-in-gel reduction).

Because TFF2 is part of the gastric mucus, it was whether TFF2 could be extracted simply by washing the gastric specimens with buffer. The resulting fraction E0 was then analyzed via SEC. Furthermore, the remaining cell pellet was extracted conventionally (fraction E1) and also analyzed via SEC (Figure 4). Surprisingly, the extract E0 (washing) contained only low-molecular-mass forms of TFF2, whereas in extract E1, TFF2 was present mainly in its high-molecular-mass form (86%) and only to lower extent in a low-molecular-mass form (13%). Both extracts contained the mucins MUC5AC and MUC6, but in different amounts (Figure 4C).

### 2.3. Binding of ^125^I-TFF2 to Human Gastric Mucin In Vitro (Overlay Assay)

**Figure 5:**
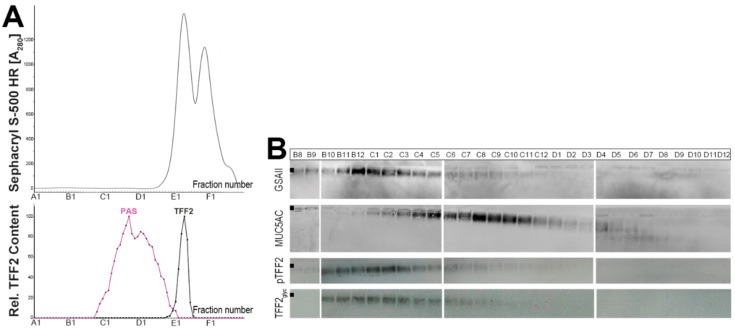
Analysis of a human gastric corpus E1-extract (M_C_-577) after reduction in boiling 1% β-mercaptoethanol. (**A**) Elution profiles after SEC on a Sephacryl S-500 High Resolution column as determined by absorbance at 280 nm. Underneath: Distribution of the relative TFF2 content. For comparison, the fractions were analyzed for their mucin content with the help of the PAS reaction (pink). (**B**) 1% AgGE and subsequent Western blot analysis of the mucin containing fractions B8-D12. The start is marked with a dot on the left. Shown are the reactivities for MUC6 (lectin GSA-II), MUC5AC, and the hybridization signals (autoradiography) obtained after incubating the blot with ^125^I-labeled porcine pancreatic TFF2 (pTFF2) or ^125^I-labeled recombinant human glycosylated TFF2 (TFF2_glyc_; overlay assays).

TFF2 has been reported to bind as a lectin to the mucin MUC6 [20]. However, separation of MUC5AC und MUC6 is not a trivial task, and it would be interesting to see if TFF2 really discriminates between MUC5AC and MUC6. Thus, we performed an *in vitro* binding study with a human gastric mucin preparation (E1-extract), which has been reduced and denatured by boiling in 1% β-mercaptoethanol. This procedure also depleted the mucin from bound TFF2. For better separation of the reduced mucin monomers, a HiPrep 16/60 Sephacryl S-500 High Resolution (S-500) column was used (Figure 5). Clearly, MUC5AC and MUC6 monomers appear to be separated (the maxima for MUC5AC and MUC6 appear in C8/C9 and B12/C1, respectively). Furthermore, both porcine, as well as recombinant human TFF2, predominantly bind to the MUC6 containing fractions.

**Figure 6:**
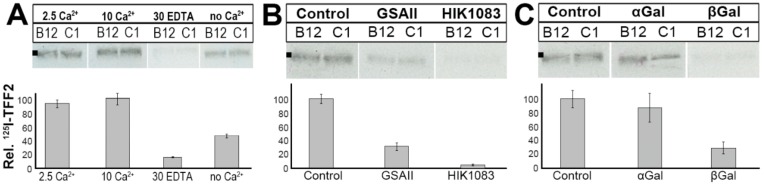
*In vitro* binding studies with ^125^I-labeled recombinant human glycosylated TFF2 (overlay assays). Human gastric mucin preparations (Mc-577) were depleted from TFF2 by reduction and purified via SEC on Sephacryl S-500 High Resolution (Figure 5). Then, the PAS-positive mucin fractions B12/C1 (Figure 5) were separated on 1% AgGE and subsequently blotted onto nitrocellulose. The start is marked with a dot on the left. Shown are the hybridization signals (autoradiography) obtained after incubating the blots with ^125^I-labeled TFF2 (overlay assay of fractions B12/C1) in the presence of 2.5 mM Ca^2+^ unless indicated otherwise. Underneath: Semi-quantitative analysis of the intensities (the relative amount of ^125^I-TFF2 bound in lanes B12/C1). (**A**) Hybridization at various Ca^2+^ concentrations: in the presence of 2.5 mM Ca^2+^, 10 mM Ca^2+^, 30 mM EDTA (and no added Ca^2+^), and no added Ca^2+^. (**B**) Hybridization after competition with the lectin GSA-II, and after competition with the antibody HIK1083. (**C**) Hybridization in the presence of 1 mM methyl-α-D-galactopyranoside (αGal), and in the presence of 1 mM methyl-β-D-galactopyranoside (βGal).

The binding of ^125^I-labeled TFF2 to TFF2-depleted human gastric mucin preparations was dependent on Ca^2+^ (Figure 6A). Addition of 2.5 mM Ca^2+^ increased TFF2 binding, whereas depletion of Ca^2+^ by addition of 30 mM EDTA reduced TFF2 binding to about 10%. Furthermore, TFF2 binding could be competitively inhibited by both the lectin GSA-II as well as the antibody HIK1083, both recognizing the terminal GlcNAc residue of a peripheral GlcNAcα1→4Galβ1→R moiety (Figure 6B). However, the monoclonal antibody HIK1083 is a much better competitive inhibitor when compared with the lectin GSA-II. We also tested whether TFF2 binding could be competitively inhibited by methyl-D-galactoside because this residue is part of the evolutionary conserved peripheral carbohydrate moiety GlcNAcα1→4Galβ1→R typical of MUC6 [21]. The β-anomer was a much better competitive inhibitor than the α-anomer (Figure 6C).

### 2.4. Binding of ^125^I-TFF2 to Porcine Gastric Mucin in Solution

**Figure 7:**
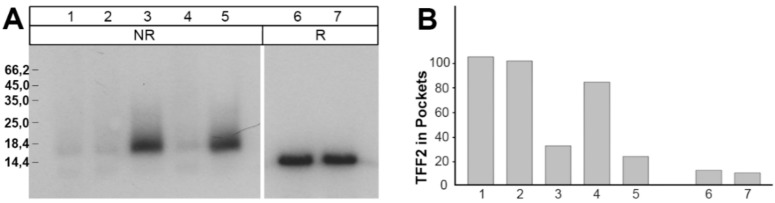
Direct binding of ^125^I-labeled recombinant human glycosylated TFF2 and a commercial porcine gastric mucin preparation (S-mucin) in solution. (**A**) ^125^I-labeled recombinant human glycosylated TFF2 was directly incubated with the S-mucin preparation, separated by 15% SDS-PAGE under non-reducing (NR) or reducing conditions (R), and the ^125^I-labeled recombinant human glycosylated TFF2 was detected by Western blotting and autoradiography. Lanes 1, 2, 4, 6, 7: mixture of ^125^I-labeled TFF2 and S-mucin; lanes 3, 5: ^125^I-labeled TFF2 without S-mucin. (**B**) The relative amount of ^125^I-labeled recombinant human glycosylated TFF2, which remained in the gel pockets after electrophoresis.

Furthermore, *in vitro* binding of TFF2 was also performed in solution where ^125^I-labeled recombinant human glycosylated TFF2 was directly mixed with a porcine gastric mucin preparation (S-mucin). This mixture of a human TFF2 and porcine mucin was then analyzed by SDS-PAGE and autoradiography (Figure 7). Clearly, under non-reducing conditions, the TFF2 did not appear as a monomer when S-mucin was present (Figure 7A; lanes 1, 2, 4); TFF2 rather did not enter the gel and was retained mainly in the gel pockets (Figure 7B). In contrast, TFF2 was detectable as a monomer when no S-mucin was present (Figure 7A; lanes 3, 5) or when the gel was run under reducing conditions (Figure 7A; lanes 6, 7). Here, TFF2 was not retained in the gel pockets (Figure 7B).

## 3. Discussion

### 3.1. TFF2 Occurs in Mucin-Bound and Low-Molecular-Mass Forms in the Human Gastric Mucosa: Differences to Porcine TFF2

As shown in Figure 1, in the human gastric mucosa, TFF2 occurs in high- and low-molecular-mass forms. The relative proportion of the two forms varies between different individuals, with the high-molecular-mass form ranging from about 65% to 95%. This seems to be a difference to the porcine system, where only the high-molecular-mass form was detectable [16,39]. The high-molecular-mass form is associated with mucins, and monomeric TFF2 (glycosylated and non-glycosylated forms; Figure 1D) can be released by boiling in SDS (Figure 1C, Figure 2 and Figure 3A) or by denaturing extraction with TRIzol^®^ (Figure 3B). Thus, human TFF2 is non-covalently bound to mucins as expected for a lectin. In contrast, in the porcine gastric mucus, the binding of TFF2 is mainly resistant to boiling in SDS and harsh extraction with TRIzol^®^ [16,39]. However, in the porcine system, non-covalent binding of TFF2 is also favored [39]. Taken together, binding of TFF2 to gastric mucin differs characteristically in human (mainly glycosylated TFF2) and pig (non-glycosylated TFF2). In the porcine gastric mucus, TFF2 is more strongly bound than in human mucus. Of note, murine Tff2, which is not glycosylated, can be released from the mucus by boiling in SDS (Znalesniak et al., manuscript in preparation) similarly as in human. Thus, we assume that it is probably the amino acid sequence, and not the glycosylation of TFF2, which determines how strong TFF2 binds.

Surprisingly, when recombinant human glycosylated TFF2 was bound to a commercial porcine gastric mucin preparation in solution, most of this recombinant TFF2 could not be released from the mucin by boiling in SDS (Figure 7). Thus far, the reason for this is not understood.

The low-molecular-mass form of TFF2 represents monomeric TFF2 (Figure 1C). Of note, under non-reducing conditions, monomeric TFF2 migrates characteristically as a somewhat higher molecular mass (Figure 1C, Figure 3C,D and Figure 4D). A possible interpretation would be that non-reduced TFF2 exists in a circular form with a disulfide bridge connecting Cys-6 and Cys-104 as shown for porcine pancreatic TFF2 [2] and this increases the hydrodynamic radius causing lower electrophoretic mobility. Furthermore, the TFF2 band under non-reducing conditions is not sharp and rather appears as a smear indicating that there might be more than one entity. An explanation would be that Cys-78 and Cys-104 are sensitive to reduction because of pairs of flanking acid amino acid residues upstream (EE and ED, respectively). Such flanking amino acid residues are known to change the pKa of cysteine residues [40,41] and could thus trigger isomerization reactions (intramolecular disulfide exchange reactions). Of note, these flanking pairs of acidic residues are conserved in human, mouse, and pig. This hypothesis is in line with the observation that the disulfide bridge between Cys-6 and Cys-104 is particularly sensitive to reduction with glutathione [42].

Furthermore, murine Tff2 also appears as a monomer, and under non-reducing conditions, part of it migrates again as a higher molecular mass as in human (Znalesniak et al., manuscript in preparation). In addition, also recombinant human glycosylated TFF2 appears as a monomer, which migrates as a higher molecular mass under non-reducing conditions (Figure 7A). This is in contrast to porcine TFF2, which exists in an unusual homodimeric form [16,39]. This homodimeric TFF2 is non-covalently linked, but resistant to boiling in SDS [16,39]. Thus, human/murine TFF2, on the one hand, and porcine TFF2, on the other hand, also differ in their property to form homodimers. Taken together, strong binding to mucins and homodimerization seem to exist in parallel. Thus, it is tempting to speculate that the strong binding of porcine TFF2 to mucin is due to the formation of unusual homodimers, which are not observed in human/mouse.

### 3.2. Soluble and Insoluble Forms of TFF2 in the Human Gastric Mucus

When gastric specimens were just washed with buffer, TFF2 was found exclusively in a low-molecular-mass form in the washings (fraction E0) (Figure 4A). Under these conditions, the mucins MUC5AC and only minor amounts of MUC6 were also released (Figure 4C), but TFF2 did not obviously bind to these mucins any more. When the remaining cell pellet was then extracted after a homogenizing step (fraction E1), high- and low-molecular-mass forms of TFF2 were detected again (Figure 4B). The high-molecular-mass fraction also contained the mucins MUC5AC and MUC6 (Figure 4C). Taken together, MUC6 mainly appears in E1, together with the high-molecular-mass form of TFF2. Thus, the association with TFF2 seems to make MUC6 less soluble, in particular when compared with MUC5AC.

These results would be in agreement with a model, where gastric mucus is composed of two layers, i.e., a loosely adherent (outer) layer and a firmly adherent, water-insoluble (inner) layer [43]. The outer layer also mixes with the gastric juice, which contains TFF2 and gastric mucins [3,14,44,45]. The concentration of TFF2 in the gastric juice shows dramatic diurnal variations [14]. In contrast, the inner layer is essential for the maintenance of the gastric mucosal pH gradient towards the acidic gastric juice [46]. It is composed of alternating layers of MUC5AC and MUC6, the latter is intermixed together with TFF2 [7,47]. Thus, TFF2 seems to fulfill an important role in stabilizing the inner gastric mucus barrier layer. This assumption is in line with the observation that *Tff2^KO^* mice showed accelerated progression to dysplasia after infection with *H. pylori* because the barrier function of gastric mucus is weakened [32].

### 3.3. TFF2 Binds to Mucin MUC6 In Vitro

When human gastric mucus was depleted of TFF2 by boiling in β-mercaptoethanol and separated by SEC on a Sephacryl S-500 column, radioactively labeled TFF2 mainly bound *in vitro* to the mucin MUC6, but not MUC5AC (overlay assay, Figure 5). This result is in agreement with previous studies, which narrowed down TFF2 binding to a peripheral GlcNAcα1→4Galβ1→4GlcNAcβ epitope typical of MUC6 [20,39]. This epitope has also been identified in human gastric mucin preparations [48].

Here, we also show that *in vitro* binding of recombinant human glycosylated TFF2 requires Ca^2+^ (overlay assay, Figure 6A). This result is in contrast to a previous report using a recombinant human TFF2 fusion protein [20] but is in agreement with *in vitro* binding studies with porcine TFF2 [39]. Furthermore, *in vitro* binding of TFF2 can be inhibited by both the lectin GSA-II and the monoclonal antibody HIK1083, but to different extents (Figure 6B). Both specifically bind to the non-reducing end of GlcNAc in carbohydrate chains, GSA-II recognizing both the α- and β-GlcNAc residues, whereas HIK1083 recognizes the α anomer only [21]. Thus, the α-GlcNAc residue is an essential part of the TFF2 recognition site. Similar results were obtained with porcine TFF2 and porcine gastric mucin [39]. Of interest, TFF2 binding can be strongly inhibited by methyl-β-Gal, but not the corresponding α anomer (Figure 6B). This is again comparable with the porcine system [39], indicating that the juxtaperipheral β-galactoside residue plays a key role the in binding of TFF2.

For the future, the question arises whether the glycoproteins DMBT1, CXCR4, and CXCR7 contain similar carbohydrate epitopes as gastric/duodenal MUC6 enabling binding of TFF2. Furthermore, it would be interesting to identify the targets of TFF2 released from cells of lymphoid organs.

## 4. Materials and Methods

### 4.1. Human Tissue

All investigations followed the tenets of the declaration of Helsinki and were approved by the Ethics Committee of the Medical Faculty of the Otto-von-Guericke University Magdeburg (codes: 01/02 January 2002 and July 2007 and 95/06 October 2006). Human tissue from the gastric corpus was investigated from 7 patients (M_C_-358, M_C_-383, M_C_-393, M_C_-405, M_C_-406A, M_C_-479, M_C_-577) undergoing gastrectomy because of carcinoma or sleeve resection because of adipositas (M_C_-577). Specimens were included in the study only when the formal histopathological review excluded neoplastic changes.

### 4.2. Extraction of Proteins

Extraction of the gastric specimens (1.6–1.8 g) with a 5-fold amount (*w/v*) of buffer (30 mM NaCl, 20 mM Tris-HCl pH 7.0 plus protease inhibitors) in a Precellys^®^24 lyser/homogenizer was performed as described in detail previously [12].

Alternatively, 0.8 g tissue was mixed with 8 mL TRIzol^®^ reagent (Ambion by Life Technologies, Darmstadt, Germany), cooled for 10 s in liquid nitrogen, and homogenized in a Precellys^®^24 lyser/homogenizer. Further extraction under these harsh denaturing conditions was according to the manufacturer’s protocol. The protein pellet was dissolved in 4 mL 1% SDS for 2 h at 50 °C.

Furthermore, a stepwise extraction protocol was also applied. Here, 1.6 g of the gastric specimens was washed with a 5-fold amount (*w/v*) of buffer (30 mM NaCl, 20 mM Tris-HCl pH 7.0 plus protease inhibitors) in a rotating wheel at 4 °C for 1 h. After centrifugation, the clear supernatant was extracted with CHCl_3_ and designated as E0. The remaining cell pellet was extracted in a Precellys^®^24 lyser/homogenizer again with a 5-fold amount of buffer, and the clear supernatant after centrifugation and extraction with CHCl_3_ was designated as E1.

### 4.3. Protein Purification by SEC

Eight milliliters of gastric extracts (or 5 mL after TRIzol^®^ extraction) was fractioned by SEC with the ÄKTA™ FPLC system (Amersham Biosciences, Freiburg, Germany) as described previously (fraction numbering: A1-A12, B1-B12, etc.) [16]. The following columns were used: HiLoad 16/600 Superdex 75 prep grade (S75HL; 20 mM Tris-HCl pH 7.0, 30 mM NaCl plus protease inhibitors; flow rate: 1.0 mL/min; 2.0 mL fractions) or HiPrep 16/60 Sephacryl S-500 High Resolution (S-500; 20 mM Tris-HCl pH 7.0, 30 mM NaCl plus protease inhibitors; flow rate: 0.5 mL/min, 2.0 mL fractions).

### 4.4. SDS-PAGE, Agarose Gel Electrophoresis, and Western Blot Analysis

Non-denaturing agarose gel electrophoresis (AgGE; containing 0.1% SDS), denaturing SDS-PAGE under reducing or non-reducing conditions, protein staining with Bio-Safe Coomassie Stain G-250 (Bio-Rad Laboratories GmbH, Munich, Germany) without fixation and periodic acid-Schiff (PAS) staining for mucins (dot blot) were described previously [15,16,49].

Western blot analyses after SDS-PAGE (electrophoretic transfer) or AgGE (capillary blot) were as reported [15,16,50]. All gels after non-reducing SDS-PAGE were subjected to post-in-gel reduction with 1% mercaptoethanol at 50 °C for 5 min before blotting as described previously [16]. Gels after AgGE were directly blotted onto nitrocellulose membranes, and for the detection with antisera, the proteins were additionally reduced on the membranes in situ with 1% mercaptoethanol at room temperature for 5 min. Both post-reduction steps enhanced sensitivity, particularly of the anti-TFF2 antiserum.

The mucins MUC5AC and MUC6 were detected with the polyclonal antiserum anti-MUC5AC-2 (1:2000 dilution) against the synthetic peptide RNQDQQGPFKMC [51] and the biotinylated lectin GSA-II from *Griffonia simplicifolia* (2 µg/mL), respectively, as reported [47,51]. TFF2 was analyzed with the affinity-purified polyclonal antiserum anti-hTFF2-2 (1:1000–4000 dilution) against the synthetic peptide FFPNSVEDCHY [39]. Affinity purification of polyclonal antisera was described in detail previously [52]. Bands were visualized with the enhanced chemiluminescence (ECL) detection system (using a secondary antibody coupled to horseradish peroxidase and luminol/p-Coumaric acid/H_2_O_2_). The chemiluminescence signal was recorded with the GeneGnome system (Syngene, Cambridge, UK) for each band within a given frame and the relative intensity was calculated (semi-quantitative analysis) using the GeneTools gel analysis software (Syngene, Cambridge, UK) setting the highest intensity in a series to 100%.

### 4.5. Digestion with Peptide-N-glycosidase F (PNGase F)

TFF2 enriched by SEC was digested with PNGase F (Roche Diagnostics GmbH, Mannheim, Germany) as described previously in detail [12].

### 4.6. TFF2 Binding Studies

Labeling of the various TFF2 peptides with ^125^I was as described previously [39]. TFF2 from the porcine pancreas, as well as recombinant human TFF2 (glycosylated and non-glycosylated forms), were kindly provided by Dr. L. Thim (Novo Nordisk A/S, Maaloev, Denmark) [53,54].

Overlay assays with ^125^I-labeled TFF2 (including hybridizations at different Ca^2+^ concentrations and competition experiments) and semi-quantitative analysis of the hybridization signals (densitometric analysis of the autoradiographies with the GeneTools software) were performed as reported previously [39]. The monoclonal antibody HIK1083 was kindly provided by Prof. H. Ota (Matsumoto, Japan) [21].

Binding of ^125^I-labeled recombinant human TFF2 (glycosylated form) in solution to a commercial porcine gastric mucin preparation (type III from Sigma-Aldrich; termed “S-mucin”) including analysis by non-reducing or reducing SDS-PAGE, Western blotting, and autoradiography was analogous as described previously [39]. Furthermore, the remaining high-molecular-mass samples not entering the gel were analyzed, as reported [39].

## 5. Conclusions

Taken together, TFF2 is an integral constituent of the human gastric mucus barrier, where it is probably a key component of the firmly attached (inner) layer due to its lectin interaction with the mucin MUC6. Binding of TFF2 in the human and porcine gastric mucosa, respectively, differ. The binding in human is much weaker, where TFF2 can be released by boiling in SDS. Furthermore, in human, no TFF2 homodimers could be detected in contrast to the pig.

## Abbreviations

AgGE	Agarose gel electrophoresis
PAS	Periodic acid-Schiff
SDS-PAGE	Sodium dodecyl sulfate-polyacrylamide gel electrophoresis
SEC	Size exclusion chromatography
TFF	Trefoil factor family

## References

[1] Tomasetto C., Rio M.C., Gautier C., Wolf C., Hareuveni M., Chambon P., Lathe R. (1990). hSP, the domain-duplicated homolog of pS2 protein, is co-expressed with pS2 in stomach but not in breast carcinoma. EMBO J..

[2] Thim L. (1997). Trefoil peptides: From structure to function. Cell Mol. Life Sci..

[3] May F.E., Semple J.I., Newton J.L., Westley B.R. (2000). The human two domain trefoil protein, TFF2, is glycosylated in vivo in the stomach. Gut.

[4] Hoffmann W., Kastin A. (2013). TFF peptides. Handbook of Biologically Active Peptides.

[5] Hanby A.M., Poulsom R., Singh S., Elia G., Jeffery R.E., Wright N.A. (1993). Spasmolytic polypeptide is a major antral peptide: Distribution of the trefoil peptides human spasmolytic polypeptide and pS2 in the stomach. Gastroenterology.

[6] Hanby A.M., Poulsom R., Elia G., Singh S., Longcroft J.M., Wright N.A. (1993). The expression of the trefoil peptides pS2 and human spasmolytic polypeptide (hSP) in ‘gastric metaplasia’ of the proximal duodenum: Implications for the nature of ‘gastric metaplasia’. J. Pathol..

[7] Ota H., Hayama M., Momose M., El-Zimaity H.M., Matsuda K., Sano K., Maruta F., Okumura N., Katsuyama T. (2006). Co-localization of TFF2 with gland mucous cell mucin in gastric mucous cells and in extracellular mucous gel adherent to normal and damaged gastric mucosa. Histochem. Cell Biol..

[8] Kouznetsova I., Kalinski T., Meyer F., Hoffmann W. (2011). Self-renewal of the human gastric epithelium: New insights from expression profiling using laser microdissection. Mol. Biosyst..

[9] Hoffmann W. (2015). Current Status on Stem Cells and Cancers of the Gastric Epithelium. Int. J. Mol. Sci..

[10] Ahnen D.J., Poulsom R., Stamp G.W., Elia G., Pike C., Jeffery R., Longcroft J., Rio M.C., Chambon P., Wright N.A. (1994). The ulceration-associated cell lineage (UACL) reiterates the Brunner’s gland differentiation programme but acquires the proliferative organization of the gastric gland. J. Pathol..

[11] Rose K., Savoy L.A., Thim L., Christensen M., Jorgensen K.H. (1989). Revised amino acid sequence of pancreatic spasmolytic polypeptide exhibits greater similarity with an inducible pS2 peptide found in a human breast cancer cell line. Biochim. Biophys. Acta..

[12] Hanisch F.G., Ragge H., Kalinski T., Meyer F., Kalbacher H., Hoffmann W. (2013). Human gastric TFF2 peptide contains an N-linked fucosylated N,N’-diacetyllactosediamine (LacdiNAc) oligosaccharide. Glycobiology.

[13] Rossez Y., Gosset P., Boneca I.G., Magalhaes A., Ecobichon C., Reis C.A., Cieniewski-Bernard C., Curt M.J.C., Leonard R., Maes E. (2014). The LacdiNAc-specific adhesin LabA mediates adhesion of *Helicobacter pylori* to human gastric mucosa. J. Infect. Dis..

[14] Semple J.I., Newton J.L., Westley B.R., May F.E. (2001). Dramatic diurnal variation in the concentration of the human trefoil peptide TFF2 in gastric juice. Gut.

[15] Kouznetsova I., Laubinger W., Kalbacher H., Kalinski T., Meyer F., Roessner A., Hoffmann W. (2007). Biosynthesis of gastrokine-2 in the human gastric mucosa: Restricted spatial expression along the antral gland axis and differential interaction with TFF1, TFF2 and mucins. Cell. Physiol. Biochem..

[16] Stürmer R., Müller S., Hanisch F.G., Hoffmann W. (2014). Porcine gastric TFF2 is a mucus constituent and differs from pancreatic TFF2. Cell. Physiol. Biochem..

[17] Hoffmann W. (2015). TFF2, a MUC6-binding lectin stabilizing the gastric mucus barrier and more. Int. J. Oncol..

[18] Thim L., Madsen F., Poulsen S.S. (2002). Effect of trefoil factors on the viscoelastic properties of mucus gels. Eur. J. Clin. Invest..

[19] Kjellev S., Nexo E., Thim L., Poulsen S.S. (2006). Systemically administered trefoil factors are secreted into the gastric lumen and increase the viscosity of gastric contents. Br. J. Pharmacol..

[20] Hanisch F.G., Bonar D., Schloerer N., Schroten H. (2014). Human trefoil factor 2 is a lectin that binds α-GlcNAc-capped mucin glycans with antibiotic activity against *Helicobacter pylori*. J. Biol. Chem..

[21] Ishihara K., Kurihara M., Goso Y., Urata T., Ota H., Katsuyama T., Hotta K. (1996). Peripheral α-linked N-acetylglucosamine on the carbohydrate moiety of mucin derived from mammalian gastric gland mucous cells: Epitope recognized by a newly characterized monoclonal antibody. Biochem. J..

[22] Ihida K., Suganuma T., Tsuyama S., Murata F. (1988). Glycoconjugate histochemistry of the rat fundic gland using *Griffonia simplicifolia* agglutinin-II during the development. Am. J. Anat..

[23] Oinuma T., Ide S., Kawano J., Suganuma T. (1994). Purification and immunohistochemistry of *Griffonia simplicifolia* agglutinin-II-binding mucus glycoprotein in rat stomach. Glycobiology.

[24] Nordman H., Davies J.R., Carlstedt I. (1998). Mucus glycoproteins from pig gastric mucosa: Different mucins are produced by the surface epithelium and the glands. Biochem. J..

[25] Lee H., Wang P., Hoshino H., Ito Y., Kobayashi M., Nakayama J., Seeberger P.H., Fukuda M. (2008). α1,4GlcNAc-capped mucin-type O-glycan inhibits cholesterol α-glucosyltransferase from *Helicobacter pylori* and suppresses *H. pylori* growth. Glycobiology.

[26] Nakayama J. (2014). Dual roles of gastric gland mucin-specific O-glycans in prevention of gastric cancer. Acta Histochem. Cytochem..

[27] Chwieralski C.E., Schnurra I., Thim L., Hoffmann W. (2004). Epidermal growth factor and trefoil factor family 2 synergistically trigger chemotaxis on BEAS-2B cells via different signaling cascades. Am. J. Respir. Cell Mol. Biol..

[28] Hoffmann W. (2004). Trefoil factor family (TFF) peptides: Regulators of mucosal regeneration and repair, and more. Peptides.

[29] Vandenbroucke K., Hans W., Van Huysse J., Neirynck S., Demetter P., Remaut E., Rottiers P., Steidler L. (2004). Active delivery of trefoil factors by genetically modified *Lactococcus lactis* prevents and heals acute colitis in mice. Gastroenterology.

[30] Baus-Loncar M., Kayademir T., Takaishi S., Wang T. (2005). Trefoil factor family 2 deficiency and immune response. Cell Mol. Life Sci..

[31] Kurt-Jones E.A., Cao L., Sandor F., Rogers A.B., Whary M.T., Nambiar P.R., Cerny A., Bowen G., Yan J., Takaishi S. (2007). Trefoil family factor 2 is expressed in murine gastric and immune cells and controls both gastrointestinal inflammation and systemic immune responses. Infect. Immun..

[32] Fox J.G., Rogers A.B., Whary M.T., Ge Z., Ohtani M., Jones E.K., Wang T.C. (2007). Accelerated progression of gastritis to dysplasia in the pyloric antrum of TFF2 -/- C57BL6 x Sv129 *Helicobacter pylori*-infected mice. Am. J. Pathol..

[33] Baus-Lončar M., Schmid J., Lalani el N., Rosewell I., Goodlad R.A., Stamp G.W., Blin N., Kayademir T. (2005). Trefoil factor 2 (TFF2) deficiency in murine digestive tract influences the immune system. Cell. Physiol. Biochem..

[34] Thim L., Mørtz E. (2000). Isolation and characterization of putative trefoil peptide receptors. Regul. Pept..

[35] Hoffmann W. (2009). Trefoil factor family (TFF) peptides and chemokine receptors: A promising relationship. J. Med. Chem..

[36] Braga Emidio N., Hoffmann W., Brierley S.M., Muttenthaler M. (2019). Trefoil factor family: Unresolved questions and clinical perspectives. Trends Biochem. Sci..

[37] Leitner V.M., Walker G.F., Bernkop-Schnurch A. (2003). Thiolated polymers: Evidence for the formation of disulphide bonds with mucus glycoproteins. Eur. J. Pharm. Biopharm..

[38] Marczynski M., Kasdorf B.T., Altaner B., Wenzler A., Gerland U., Lieleg O. (2018). Transient binding promotes molecule penetration into mucin hydrogels by enhancing molecular partitioning. Biomat. Sci..

[39] Stürmer R., Harder S., Schlüter H., Hoffmann W. (2018). Commercial porcine gastric mucin preparations, also used as artificial saliva, are a rich source for the lectin TFF2: In vitro binding studies. ChemBioChem.

[40] Gilbert H.F., Meister A. (1990). Molecular and cellular aspects of thiol-disulfide exchange. Advances in Enzymology and Related Areas of Molecular Biology.

[41] Poole L.B. (2015). The basics of thiols and cysteines in redox biology and chemistry. Free Radical Biol. Med..

[42] Otto W.R., Rao J., Cox H.M., Kotzian E., Lee C.Y., Goodlad R.A., Lane A., Gorman M., Freemont P.A., Hansen H.F. (1996). Effects of pancreatic spasmolytic polypeptide (PSP) on epithelial cell function. Eur. J. Biochem..

[43] Johansson M.E., Sjovall H., Hansson G.C. (2013). The gastrointestinal mucus system in health and disease. Nat. Rev. Gastroenterol. Hepatol..

[44] Kubota S., Yamauchi K., Sugano M., Kawasaki K., Sugiyama A., Matsuzawa K., Akamatsu T., Ohmoto Y., Ota H. (2011). Pathophysiological investigation of the gastric surface mucous gel layer of patients with *Helicobacter pylori* infection by using immunoassays for trefoil factor family 2 and gastric gland mucous cell-type mucin in gastric juice. Dig. Dis. Sci..

[45] Hanisch F.G., Chai W., Rosankiewicz J.R., Lawson A.M., Stoll M.S., Feizi T. (1993). Core-typing of O-linked glycans from human gastric mucins. Lack of evidence for the occurrence of the core sequence Gal1-6GalNAc. Eur. J. Biochem..

[46] Phillipson M., Atuma C., Henriksnas J., Holm L. (2002). The importance of mucus layers and bicarbonate transport in preservation of gastric juxtamucosal pH. Am. J. Physiol. Gastrointest. Liver Physiol..

[47] Ota H., Katsuyama T. (1992). Alternating laminated array of two types of mucin in the human gastric surface mucous layer. Histochem. J..

[48] Rossez Y., Maes E., Lefebvre Darroman T., Gosset P., Ecobichon C., Curt M.J.C., Boneca I.G., Michalski J.C., Robbe-Masselot C. (2012). Almost all human gastric mucin O-glycans harbor blood group A, B or H antigens and are potential binding sites for *Helicobacter pylori*. Glycobiology.

[49] Albert T.K., Laubinger W., Müller S., Hanisch F.G., Kalinski T., Meyer F., Hoffmann W. (2010). Human intestinal TFF3 forms disulfide-linked heteromers with the mucus-associated FCGBP protein and is released by hydrogen sulfide. J. Proteome Res..

[50] Jagla W., Wiede A., Kölle S., Hoffmann W. (1998). Differential expression of the TFF-peptides xP1 and xP4 in the gastrointestinal tract of *Xenopus laevis*. Cell Tissue Res..

[51] Kouznetsova I., Peitz U., Vieth M., Meyer F., Vestergaard E.M., Malfertheiner P., Roessner A., Lippert H., Hoffmann W. (2004). A gradient of TFF3 (trefoil factor family 3) peptide synthesis within the normal human gastric mucosa. Cell. Tissue Res.

[52] Wiede A., Jagla W., Welte T., Köhnlein T., Busk H., Hoffmann W. (1999). Localization of TFF3, a new mucus-associated peptide of the human respiratory tract. Am. J. Respir. Crit. Care Med..

[53] Thim L., Norris K., Norris F., Nielsen P.F., Bjorn S.E., Christensen M., Petersen J. (1993). Purification and characterization of the trefoil peptide human spasmolytic polypeptide (hSP) produced in yeast. FEBS Lett..

[54] Jørgensen K.H., Thim L., Jacobsen H.E. (1982). Pancreatic Spasmolytic Polypeptide (PSP): I. Preparation and initial chemical characterization of a new polypeptide from porcine pancreas. Regul. Peptides.

